# Multiple ASC-dependent inflammasomes drive differential pro-inflammatory cytokine production in a mouse model of tendinopathy

**DOI:** 10.1042/BSR20241282

**Published:** 2024-11-21

**Authors:** Alejandro Peñín-Franch, Laura Hurtado-Navarro, José Antonio García-Vidal, Pilar Escolar-Reina, Francesc Medina-Mirapeix, Pablo Pelegrin

**Affiliations:** 1 Molecular Inflammation and Physiotherapy gropus, Biomedical Research Institute of Murcia (IMIB), 30120 Murcia, Spain; 2 Department of Physical Therapy, University of Murcia, 30120 Murcia, Spain; 3 Department of Biochemistry and Molecular Biology B and Immunology, University of Murcia, 30120 Murcia, Spain

**Keywords:** ASC, Collagenase, inflammasome, NLRP3, sterile tissue damage, tendon

## Abstract

Inflammasomes are multiprotein complexes that regulate the bioactive production of IL-1β and IL-18, being implicated in the inflammatory response of different diseases. The inflammasome formed by the cytosolic sensor NLRP3 is highly promiscuous, as it could be activated by different pathogen- and sterile-signals. However, few models have studied the implication of NLRP3 in tissue damage-induced inflammation, particularly the implication of NLRP3 in tendinopathies. Here, we aimed to investigate the implication of NLRP3 in a mouse model of tendinopathy by collagenase degradation of the extracellular matrix in the Achilles’ mice tendon. We found that NLRP3 was involved in the production of IL-1β, but another ASC-dependent inflammasome was required to produce IL-18 during sterile tissue damage. Our study suggests that in the immune response to extracellular matrix degradation different inflammasomes, probably expressed in different cell compartments, were able to differentially control IL-1β and IL-18 production *in vivo*. These results suggest the potential use of therapies targeting ASC as beneficial in the treatment of tendinopathies.

## Introduction

The inflammasome is a multiprotein complex that facilitates the activation of caspase-1 and the subsequent production of mature pro-inflammatory cytokines interleukin (IL)-1β and IL-18 after proteolytically cleavage [1]. Different inflammasome sensor proteins are triggered in different cells in response to different activators, most of them belong to the nucleotide-binding oligomerization domain and leucine-rich repeat receptor (NLR)-family of proteins. Upon activation, most of the NLRs induce the oligomerization of the inflammasome adaptor protein ASC (Apoptosis-associated speck-like protein containing a caspase activation domain) to recruit and activate caspase-1 [2]. Meanwhile, all inflammasomes could be triggered in response to different pathogens, NLRP1 and NLRP3 inflammasomes could be also activated upon recognition of endogenous host-related danger signals [2]. While human NLRP1 expression is restricted to epithelial cells, NLRP3 is mainly expressed in myeloid cells. NLRP3 forms the most promiscuous inflammasome since it could be triggered by a wide range of stimuli, such as particulate matter, crystals, or extracellular ATP, as well as extracellular matrix degradation [2–4]. Almost all of the different NLRP3 activators result in a decrease of intracellular K^+^ that changes NLRP3 conformation towards an active oligomeric complex [5,6]. The NLRP3 inflammasome has been implicated in the pathophysiological process of several chronic inflammatory, metabolic, and degenerative diseases [7], including several models of sterile inflammation induced by uric acid crystals, silica particles, or Alum [5,8,9]. Injuries of tendons in the food and ankle by overuse are the most common type of tendinopathies, constituting in some cases a chronic injury [10]. The pathogenesis of tendinopathies includes degradation of extracellular matrix and inflammation [11,12], however, the role of the NLRP3 inflammasome has not being studied in the context of tendinopathies. In this study, we investigated the implication of NLRP3 in an *in vivo* model of tendinopathy, by inducing a sterile tendon damage injecting collagenase in the Achilles’ mice tendon and found that while NLRP3 controls IL-1β production, other ASC-dependent inflammasome was required for IL-18 production. Our study potentially proposes a beneficial use of ASC blockers to treat tendinopathies-associated inflammation.

## Methods

### Animals and procedures

Mice procedures were approved by the University of Murcia ethical committee and the Animal Health Service of the General Directorate of Fishing and Farming of the Council of Murcia (#A13160702). C57/BL6 mice were obtained from Jackson Laboratories, and *Nlrp3*^−/−^ and *Pycard*^−/−^ in C57/BL6 background were already described [13]. Mice were bred in specific pathogen-free conditions at the animal house of the *Hospital Virgen Arrixaca* with a 12:12 h light–dark cycle and used between 8 and 10 weeks of age. Sterile tissue damage was performed at the animal house of the University of Murcia with an injection of 20 µl of collagenase A (10 µg/µl, Sigma–Aldrich) on one paw of isoflurane-anesthetized mouse. The other paw was either non-injected or injected with 20 µl of saline solution and was used as a control. Different days after injection (from 1 to 21) the animals were sacrificed with CO_2_ inhalation and the calcaneal tendon was dissected for RT-qPCR studies, or the zone between gastrocnemius and calcaneus, including the tendon, adipose tissue, tibia and peroneus, was dissected for ELISA and histology.

### Cytokine evaluation

Tendons were homogenized in homobuffer (70 mM saccharose, 220 mM mannitol, 2 mM Tris-HCl, 0.1 mM EDTA, 0.1% bovine serum albumin, and pH = 7.4, supplemented with protease inhibitors) and were used in ELISA for mouse IL-1β (high-sensitivity, Invitrogen), following the manufacturer’s instructions and read in a Synergy Mx plate reader (BioTek). Multiplexing for IL-6, IL-18, TNFα, and CXCL10 was performed using the Luminex colour-coded antibody-immobilized beads from Invitrogen, and the results were analyzed in a Luminex MAGPIX instrument (Luminex Corporation).

### Quantitative reverse transcriptase-polymerase chain reaction (RT-qPCR) analysis

Tendons were homogenized using an Omni THQ homogenizer in Qiazol (Qiagen). Then left for 5 min at room temperature and centrifuged at 12000 × *g* for 15 min at 4°C. The upper phase was mixed with one volume of 70% ethanol and loaded in RNeasy Mini Kit columns (Qiagen). Total RNA was isolated after DNase I (Qiagen) treatment following the manufacturer’s instructions. Reverse transcription was performed using the iScript cDNA Synthesis kit (BioRad). The mix SYBR Green Premix ExTaq (Takara) was used for qPCR in an iCyclerMyiQ thermocycler (BioRad). Specific primers were from Sigma (KiCqStart SYBR Green Primers) and relative expression of *Il6* and *Il1b* was normalized to the housekeeping gene *Actb* using the 2^−ΔCt^ method.

### Histopathology

Mice paws were fixed in 4% p-formaldehyde (Sigma–Aldrich) for 24 h, processed, paraffin-embedded, and sections stained with hematoxylin and eosin. Quantification of polymorphonuclear cells was performed by nuclear morphology in an AxioScope AX10 microscope (Carl Zeiss) and pictures were taken with an AxioCam506 Color (Carl Zeiss).

### Statistics

Statistical analyses were performed using Prism (GraphPad). Data were transformed using Log_2_ for statistical analysis. Unpaired *t*-test was used for comparisons of two groups and two-way ANOVA for two variable comparisons between different groups. The test used in each panel is mentioned in the figure legend. Data are shown as mean values and error bars represent standard error from the number of independent assays indicated in the figure legend, which are also overlaid in the histograms as dots. *S*ignificant differences were annotated as ****P<0.0001, ***P<0.001, **P<0.01, *P<0.05, *ns* for *P*>0.05, not significant.

## Results

**Figure 1:**
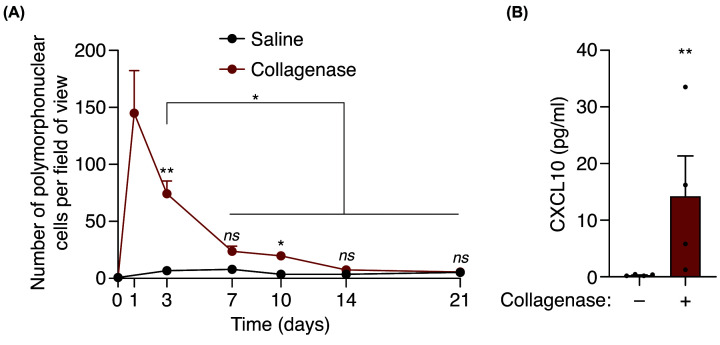
Collagenase-induced tissue damage provokes a local inflammatory response (**A**) Quantification of polymorphonuclear cells per field of view of calcaneal tendon sections treated or not with collagenase for the indicated time. Centre values represent the mean (*n* = 3–15 independent animals per time point) and error bars represent s.e.m. Two-way ANOVA, *P<0.05, **P<0.005, *ns P*>0.05. (**B**) CXCL10 detection from calcaneal tendons of wild-type mice treated or not with collagenase after 3 days. Centre values represent the mean (*n* = 4 independent animals) and error bars represent s.e.m. Unpaired *t*-test, **P<0.005.

We initially examined the infiltration of polymorphonuclear (PMN) cells in the periphery of the tendon after extracellular matrix degradation by collagenase. PMN cells peaked at 1 day after local collagenase administration and decreased afterwards, reaching basal levels after 14 days (Figure 1A). This result shows that the inflammatory response was resolved within 1 month after local collagenase administration. After collagenase injection, we also found an increase in the chemokine CXCL10 (Figure 1B), suggesting a potential chemoattractant function of this chemokine towards PMN to injury.

**Figure 2:**
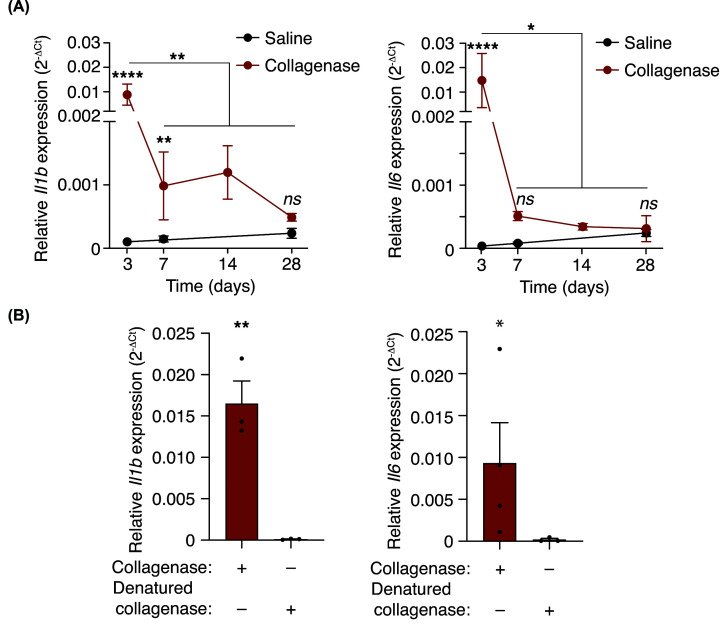
Tissue damage provokes an elevation of IL-1β and IL-6 (**A,B**) Quantitative PCR for *Il1b* and *Il6* in the calcaneal tendons of wild-type mice after different times of collagenase injection as indicated (**A**) or after 3 days of denatured collagenase injection (**B**). Centre values represent the mean (*n* = 3–6 independent animals) and error bars represent s.e.m.; Two-way ANOVA (**A**) or unpaired *t*-test (**B**) ****P<0.0001, **P<0.01, *P<0.05, and *ns P*>0.05.

After tissue injury, we also found an increase in the expression of the pro-inflammatory cytokines *Il1b* and *Il6* (Figure 2A). The induction of *Il1b* and *Il6* decreased with the time after collagenase administration (Figure 2A) and was not observed when denatured collagenase was used (Figure 2B). Therefore, collagenase activity, probably by degradation of the extracellular matrix, was required to induce sterile tissue damage driven by an inflammatory infiltrate of PMN cells and an increase in pro-inflammatory cytokines.

**Figure 3:**
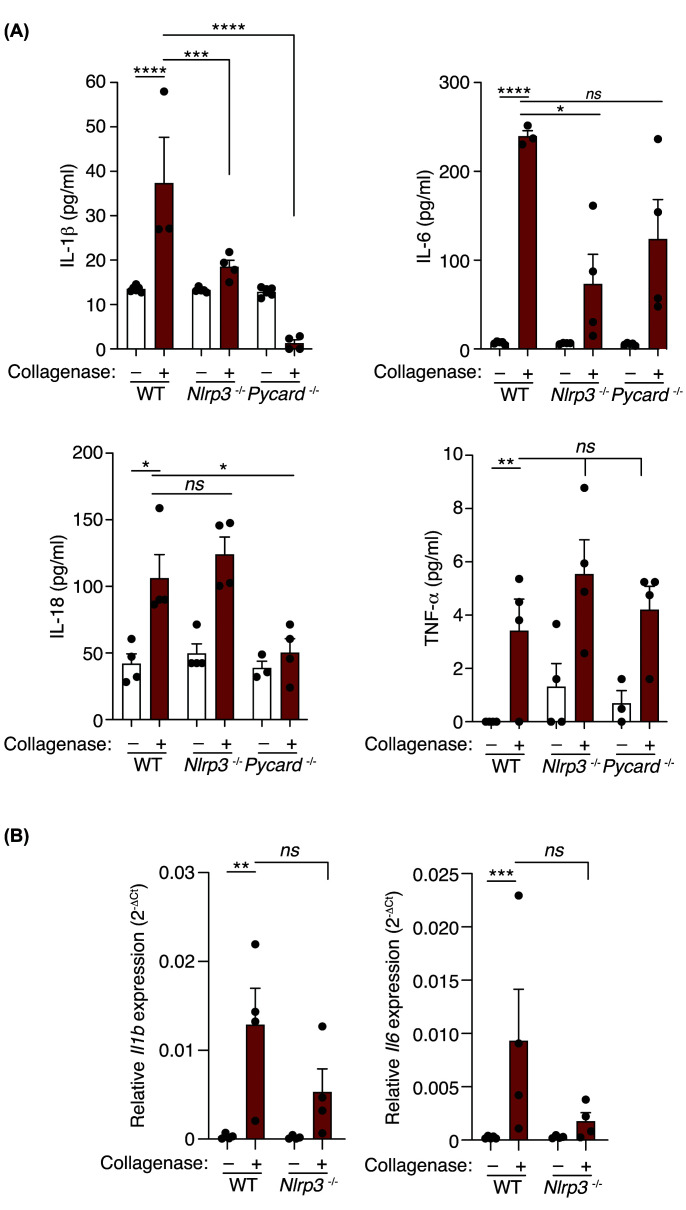
Pro-inflammatory cytokine production in response to collagenase-induced tissue damage is controlled by different ASC-dependent inflammasomes (**A**) IL-1β, IL-6, IL-18, and TNF-α detection in calcaneal tendons of wild type, *Nlrp3*^−/−^ and *Pycard*^−/−^ mice treated or not with collagenase for 3 days. Centre values represent the mean (*n* = 3–6 independent animals) and error bars represent s.e.m.; Two-way ANOVA, ****P<0.0001, ***P<0.001, **P<0.01, *P<0.05, *ns P*>0.05. (**B**) Quantitative PCR for *Il1b* and *Il6* in the calcaneal tendons of wild type and *Nlrp3*^−/−^ mice treated as in (**A**). Centre values represent the mean (*n* = 4–5 independent animals) and error bars represent s.e.m.; Two-way ANOVA, ***P<0.001, **P<0.01, *ns P*>0.05.

We next aimed to assess if the inflammatory response induced by collagenase was dependent on the NLRP3 inflammasome and found that both IL-1β and IL-6 were reduced in NLRP3-deficient mice (Figure 3A). However, IL-18 and TNFα were not affected in the *Nlrp3*^−/−^ mice (Figure 3A), but *Il1b* and *Il6* gene expression was reduced in tendons of the *Nlrp3*^−/−^ mice (Figure 3B). To confirm a role for active inflammasomes and assess the NLRP3-independent production of IL-18, we used mice deficient in the common inflammasome adaptor protein ASC. Collagenase administration in *Pycard*^−/−^ mice resulted in a reduction of IL-1β, IL-6, and IL-18, but not of TNFα (Figure 3A). This suggests that IL-18 production after tissue injury is dependent on the activation of an ASC-inflammasome.

**Figure 4:**
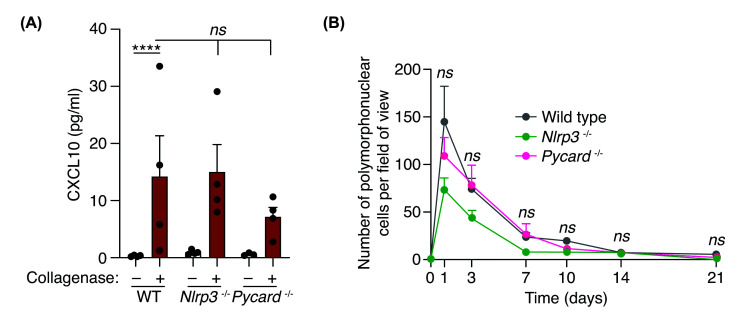
Polymorphonuclear cell infiltration is independent on ASC-dependent inflammasomes (**A**) CXCL10 detection from calcaneal tendons of wild type, *Nlrp3*^–/–^ and *Pycard*^–/–^ mice treated or not with collagenase for 3 days. Centre values represent the mean (*n* = 3–4 independent animals) and error bars represent s.e.m.; Two-way ANOVA, ****P<0.0001, *nsP*>0.05. (**B**) Quantification of polymorphonuclear cells per field of view of calcaneal tendons sections of wild type, *Nlrp3*^–/–^ and *Pycard*^–/–^ mice treated as in (**A**) but for different times as indicated. Centre values represent the mean (*n* = 2–15 independent animals per time point) and error bars represent s.e.m.; Two-way ANOVA, *nsP*>0.05.

We finally examined if the inflammasome was affecting the production of the chemokine CXCL10 and the infiltration of PMN cells into the tissue, however, neither of these two parameters was significantly affected (Figure 4A,B) and the resolution of the inflammation, in terms of PMN cell infiltration, was neither significantly affected by the lack of NLRP3 or ASC (Figure 4B). Therefore, while inflammasomes control pro-inflammatory cytokine production during sterile tissue injury, it did not affect TNF-α, chemokine production or PMN infiltration.

## Discussion

In this study, we found that NLRP3 controls the production of IL-1β in a mouse model of tendinopathy by sterile extracellular matrix degradation, while an alternative ASC-dependent inflammasome controls IL-18 production. This result is surprising as IL-18 is a cytokine depending on caspase-1 activation for maturation and release [1], and although IL-6 is not an inflammasome-dependent cytokine, it is known that IL-1β blocking reduces the production of IL-6 in different pathologies [14]. Therefore, a reduction of IL-1β production in the *Nlrp3*^−/−^ mice could be controlling IL-6 production in this *in vivo* model of sterile tissue injury. However, meanwhile ASC deficiency also reduce IL-1β production, IL-6 was not affected in the *Pycard*^−/−^ mice. Therefore, that degradation of extracellular matrix could be inducing different ASC-dependent inflammasomes, that could be expressed in different cell types, being the production of IL-18 dependent from a non-NLRP3 but ASC-dependent inflammasome. While IL-1β is mainly produced by myeloid cells expressing NLRP3, IL-18 could be largely produced by epithelial cells that do not express NLRP3, but rather express NLRP1 and/or NLRC4 [2,15,16]. For example, autoinflammatory syndromes with gain-of-function mutations in NLRP3 are mainly driven by myeloid-production of IL-1β, while syndromes due to gain-of-function mutations in NLRC4 are associated with epithelial-production of IL-18 [17,18]. Also, IL-1β and IL-18 expression present different regulatory mechanisms [19], that could explain its differential production in different cells. However, differential production of IL-1β and IL-18 has not been always dissociated in *in vivo* models, and in allogenic transplantation induced a local production of both cytokines dependent on NLRP3 [20]. Therefore, our results confirm that in tendinopathies, differential IL-1β and IL-18 production could be controlled by different inflammasomes.

The NLRP3 inflammasome has been implicated in different diseases where the inflammatory response has an important role, being pharmacological targeting of NLRP3 an important area of development for the clinical treatment of different inflammatory conditions [7,21]. The results of our study propose that pharmacologic treatment of the inflammatory response associated to tendinopathy would be ideally treated with pan-inflammasome inhibitors [22] or therapies targeting ASC [23,24], rather than specific NLRP3 blockers or other inhibitors targeting single inflammasomes. This might be important for the clinical application of NLRP3 blockers in multi-factorial complex chronic diseases where the NLRP3 inflammasome is one of the contributing pathways [7,21].

Our study found that the inflammatory response driving the infiltration of PMN cells in our tendinopathy model after tendon injury was independent of ASC-dependent inflammasomes. This is in line with previous studies showing that in the absence of NLRP3, the application of galvanic currents in the mice tendon to activate NLRP3 did not affect the infiltration of PMN cells [13]. In contrast, another study shows that necrotic cells injected in the peritoneum of mice induces an NLRP3-dependent IL-1 production and control PMN cell infiltration into the peritoneum [25]. Also, peritoneal administration of uric acid crystals, Alum or silica particles, also induce an NLRP3-dependent PMN infiltration [5,8,9]. These differences could be due to the activation of different signalling pathways in the different models, the intraperitoneal or intratracheal administration of Alum or silica results in an inflammasome-dependent response mostly dependent on IL-1, since PMN infiltration depends on IL-1R signaling [8,9]. In our study there is a production of TNF-α and CXCL10 independently on the inflammasome, therefore CXCL10 could be recruiting PMN cells. Similarly, the effect of necrotic cells was dependent on ATP-derived from mitochondria of necrotic cells acting as a damage-associated signal that mainly activate the NLRP3 inflammasome [25]. In our tendinopathy model, we do not know the exact signal controlling NLRP3 and potentially other ASC-dependent inflammasomes. We could assume that *in vivo*, extracellular matrix degradation by collagenase could induce the presence of several damage-associated signals, as exposure of primed macrophages to collagenase-degraded collagen was unable to activate the inflammasome (not shown). ATP or other transient released damaged signals, as well as production of inflammatory mediators independent of the inflammasome (i.e. TNFα) might function as priming and activating signals for NLRP3 and other inflammasomes *in vivo* [26].

In conclusion, we found that the NLRP3/ASC-inflammasome controls the production of IL-1β, meanwhile, another ASC-dependent inflammasome is required to produce IL-18 in tendinopathy induced by sterile tissue degradation of the extracellular collagen matrix. However, the production of TNFα and CXCL10 chemokine and transient PMN cell infiltration in tendinopathies is independent of ASC inflammasomes. The understanding of the specific inflammatory microenvironment in tendinopathies will help to the development of novel pharmacological treatments.

## Data Availability

The raw data supporting the conclusions of this article will be made available by the authors, without undue reservation.

## Abbreviations

ASC: Apoptosis-associated speck-like protein containing a caspase activation domain
IL: interleukin
NLR: nucleotide-binding oligomerization domain and leucine-rich repeat receptor
PMN: Polymorphonuclear cells
TNFα: Tumor necrosis factor alpha
